# Analysis of lipoprotein SPD_0792 from *Streptococcus pneumoniae* as a potential vaccine candidate

**DOI:** 10.6026/973206300211784

**Published:** 2025-07-31

**Authors:** Shabnam Shabnam, Kaur Kaur, Devinder Sehgal

**Affiliations:** 1 Molecular Immunology Laboratory, National Institute of Immunology, Aruna Asaf Ali Marg, New Delhi, India

**Keywords:** *Streptococcus pneumoniae*, pneumococcal protein vaccine, lipoprotein, SPD_0792

## Abstract

*Streptococcus pneumoniae* remains a major global health threat, especially in young children and the elderly. Although vaccines are available, polysaccharide-based (serotype-specific) formulations are limited by high costs and rising serotype replacement. As a result, there is growing interest in developing a broadly protective, protein-based vaccine. Therefore, it is of interest to investigate SPD_0792, a putative lipoprotein, as a potential vaccine candidate. Sequence analyses revealed high conservation and surface localization. The presence of multiple B-cell and helper T-cell epitopes in SDP_0792 were identified. Structural modelling and refinement using the GalaxyRefine server confirmed a stable fold comprising of 38.9% α-helices. SPD_0792 was successfully cloned, expressed and partially purified. These findings support the potential of SPD_0792 as a novel, serotype-independent antigen for next-generation pneumococcal vaccines.

## Background:

*Streptococcus pneumoniae* is an encapsulated bacterium that asymptomatically colonizes the human nasopharynx but can cause invasive disease such as pneumonia, sepsis and meningitis [1]. It remains a leading cause of morbidity and mortality worldwide, particularly among infants (<2 years) and the elderly [2]. The burden of pneumococcal disease is disproportionately high in low- and middle-income countries, where limited access to healthcare and overcrowding contribute to transmission. In many regions of Asia and sub-Saharan Africa, childhood pneumonia and meningitis due to *S. pneumoniae* rank among the top causes of death, underscoring an urgent need for improved preventive measures [3]. Available polysaccharide-based pneumococcal vaccines have significantly reduced disease in vaccinated populations but have important limitations. The 23-valent pneumococcal polysaccharide vaccine (PPV23) and the various conjugate vaccines (PCV10, PCV13 and newer formulations) target a subset of the about 100 known serotypes [4]. By concentrating on a restricted set of serotypes, these vaccines neglect many other serotypes, thereby facilitating the emergence of non-vaccine serotypes (serotype replacement) and perpetuation of disease transmission. Moreover, unconjugated polysaccharide vaccine is poorly immunogenic in infants and induces T-independent responses that lack immunological memory. Conjugate vaccines overcome this in young children but are expensive to produce, requiring multiple doses and cold-chain logistics. In practice, vaccine coverage can be incomplete and efficacy may wane with age or in immunocompromised individuals [5]. These factors contribute to persistent pneumococcal disease in settings with limited vaccine access and underscore the need for alternative strategies. A protein-based pneumococcal vaccine offers a serotype-independent approach with the potential for broad protection across diverse strains and age groups [6]. Unlike polysaccharide capsules, many pneumococcal surface proteins are highly conserved among serotypes. By targeting antigens such as pneumolysin [7] and pneumococcal surface protein A (PspA) [8], a protein vaccine can elicit immune responses that recognize all serotypes, including those not covered by current polysaccharide-based pneumococcal vaccine formulations. Researchers have also tested cocktails of 2 or more protein antigens in mouse model of pneumococcal infection for their ability to confer protection. Verhoeven and colleagues showed that immunization with a trivalent (PcpA, PlyD1 and PhtD) protein vaccine candidate confers protection in an infant murine model of lethal pneumococcal pneumonia [9]. Vaccination with a combination of pneumococcal virulence proteins PspC, PdB and PspA has been demonstrated to be protective against invasive pneumococcal disease [10].

Lipoproteins are an attractive class of surface proteins that have drawn the attention of researchers as potential vaccine candidates [11]. In Gram-positive bacteria such as *S. pneumoniae*, the mature lipoprotein is membrane-anchored via its N-terminal lipidated cysteine, with the protein portion extending into the extracellular environment [12]. Lipoproteins PiaA and PiuA, and SP0845 have been shown to confer protection in mouse challenge experiments [13, 14] because, protein subunit vaccines are generally easier to manufacture and more stable, they may also offer logistical and cost advantages for global immunization programs. These attributes make protein-based vaccines an attractive next-generation strategy to overcome the serotype dependence and coverage gaps of existing pneumococcal vaccines [4, 5- 6]. Recent advances in computational biology have accelerated the identification of protein vaccine candidates. Immunoinformatic tools such as ABCpred and IEDB analysis resource are being used to scan protein sequences for B-cell and T-cell epitopes that are likely to be strongly immunogenic and conserved across strains. Mir and coworkers have identified T-cell and B-cell epitopes in the RNA-dependent RNA polymerase of SARS-CoV-2 [15]. Predicted epitopes that bind broadly to MHC alleles guided the selection of peptide regions for inclusion in vaccine constructs. Once candidate proteins or epitopes were identified, three-dimensional (3D) modelling helped in assessing the structural stability and surface accessibility of antigenic regions. Using these *in silico* approaches, novel multi-epitope subunit vaccines have been designed for *Acinetobacter baumannii* [16], *Mycobacterium tuberculosis* [17] and coxsackievirus B [18] and *Toxoplasma gondii* [19].

Despite advances in vaccination, *S. pneumoniae* continues to cause significant disease, particularly in vulnerable populations. Existing vaccines are costly and serotype-specific. Therefore, it is of interest and urgent need for affordable, serotype-independent protein-based pneumococcal vaccines. A key step in this approach is the identification of surface-exposed, immunogenic proteins that are conserved across strains and capable of inducing both B-cell and T-cell responses. In this study, we performed a genome-wide analysis of *S. pneumoniae* strain D39 to identify such candidates. Among the proteins identified, a putative lipoprotein SPD_0792, was selected for further characterization. We analysed its cellular localization, structure and immunogenicity using in silico tools, and purified recombinant (rSPD_0792).

## Materials and Methods:

## Signal peptide prediction:

The full-length amino acid sequence of SPD_0792 (accession number ABJ54401) from *S. pneumoniae* strain D39 was downloaded from the NCBI. The protein domain and architecture were identified using the Conserved Domain Database. The architecture of the operon encoding SPD_0792 was assessed using Operon mapper [20]. The presence of signal peptide in SPD_0792 was determined by using SignalP 6.0 [21].

## Secondary structure prediction and 3D modelling:

In order to gain insights into structure and function of SPD_0792 its secondary and 3D structure was modelled. Secondary structural elements of SPD_0792 were predicted using PSIPRED [22]. For the 3D homology model of SPD_0792 we searched SWISS-MODEL and Protein Database PDB [23]. The quality of the model was evaluated using the SWISS-MODEL structure assessment tool. The model was further refined using GalaxyRefine server [24]. Additionally, structural validation and error estimation were performed using the ProSA server [25].

## B-cell and helper T-cell epitope prediction:

Linear (continuous) B-cell epitopes were predicted using ABCpred and BCPred servers with a threshold cutoff score of 0.75 and a window size of 16 amino acids [26]. Conformational (discontinuous) epitopes were predicted using ElliPro, which assigns a protrusion index (PI) score to each predicted epitope based on protein 3D structure [27]. To predict MHC class II-restricted T-cell epitopes, the IEDB analysis resource was used [28]. The alleles that are expressed in mouse were taken into consideration for predicting MHC class II-restricted T-cell epitope. Predictions were performed using the IEDB-recommended method, yielding 15-mer peptides ranked by percentile score.

## Molecular cloning, expression and partial purification of rSPD_0792:

The spd_0792 gene fragment (amino acids 23-290, excluding the signal peptide) was amplified from *S. pneumoniae* D39 genomic DNA using primers DS_2033 (ccccccgaattcgcaacaacaacatgctacttctgag; sense) and DS_2034 (ccccccctcgagaagtttaacccacttatcattatcc; antisense). EcoRI (in DS_2033) and XbaI (in DS_2034) restriction sites were incorporated in the primers to facilitate cloning. The PCR product was digested with EcoRI and XbaI, and cloned into similarly digested pET-22b(+) expression vector (Novagen, USA). The recombinant plasmid was transformed into *E. coli* strain DH5α, and confirmed by restriction enzyme digestion and DNA sequencing. For protein expression, the construct was transformed into *E. coli* strain BL21 (DE3). Expression conditions were optimized by varying induction temperature and IPTG concentration to obtain soluble rSPD_0792. Following optimization, rSPD_0792 was purified from the soluble fraction as a C-terminal His-tagged protein using Ni-NTA affinity chromatography (Qiagen, USA). Protein purity was assessed by SDS-PAGE.

## Immunoblotting:

The purified protein fraction was resolved on SDS-PAG (12%) and transferred onto a nitrocellulose membrane using a wet transfer system (Bio-Rad, USA). Transfer was performed at 120 mA for 12 h at 4°C. The membrane was blocked with 5% (w/v) BSA in PBS for 1h at room temperature and then washed three times with PBST. The blot was incubated with primary anti-His antibody (1:5000 dilution; Southern Biotech, USA) for 1 h, followed by HRP-conjugated goat anti-mouse IgG (1:10,000 dilution; Southern Biotech). After washing, signal was developed using ECL and visualized on a gel documentation system.

## Ethics statement:

The research was conducted in accordance with the guidelines and regulations approved by the Institutional Biosafety Committee of the National Institute of Immunology (IBSC#415/20).

## Results and Discussion:

BLASTP homology search of the non-redundant database at NCBI and Conserved Domain Database revealed that SPD_0792 has a domain of unknown function DUF4300 (pfam14133). Operon analysis indicated that spd_0792 is encoded in the *S. pneumoniae* D39 genome as a monocistronic operon. SPD_0792 is highly conserved across pneumococcal strains. It showed an amino acid identity of 95.4 to 100% in 245 pneumococcal strains analysed, suggesting that it plays an important role in pneumococcal biology. Analysis of SPD_0792 sequence using SignalP 6.0 identified an N-terminal signal sequence terminating at a conserved cysteine residue within a canonical lipobox motif [LVI] [ASTVI] [GAS] [C], characteristic of bacterial lipoproteins. The mature lipoprotein is anchored in the membrane through the lipidated cysteine. The C-terminal part of the lipoprotein is exposed to the external milieu and can be targeted by the humoral immune system [11]. Although the precise biological function of SPD_0792 remains to be elucidate our findings indicate that it is a surface-exposed lipoprotein. In various bacterial pathogens, surface lipoproteins have been implicated in key processes such as adhesion, nutrient acquisition, colonization, metabolic fitness and virulence/ pathogenesis [11, 29, 30, 31]. Given these established roles, further investigations are warranted to elucidate whether SPD_0792 contributes to similar mechanisms in *S. pneumoniae*.

Secondary structure prediction using PSIPRED revealed that SPD_0792 consists of 38.9% α-helices (113 residues), 14.8% β-strands (43 residues) and 46.2% random coils (134 residues). We searched the PDB for possible matches to SPD_0792. No results matching the query sequence was found. The PDB (with CSM feature) and the SWISS-MODEL server, however, showed a 100% match with a computer structure model of the homologous protein from *S. pneumoniae* strain R6, an avirulent derivative of pneumococcal strain D39 (AlphaFold DB: AF-Q8CYX1-F1) with a predicted local distance difference test [pLDDT (global)] score of 88.94. pLDDT is a per-residue metric that reflects the local confidence of a structural prediction. It ranges from 0 to 100, with higher values indicating greater confidence and generally higher prediction accuracy. Scores above 90 suggest that both the backbone and side chains are predicted with high reliability. Model validation using a Ramachandran plot showed that 88.19% of residues were in favoured regions, with only 2.43% in disallowed regions. Further, refinement of the model using GalaxyRefine server (Figure 1A - see PDF) improved the score to 98.26% (Figure 1B - see PDF). Additionally, the refined model had a ProSA z-score of -7.49, indicating good structural quality and stability (Figure 1C - see PDF).

The primary mechanism of clearance of *S. pneumoniae* from the host is by opsonophagocytosis. For protein antigens like SPD_0792, the host mounts a T-cell dependent antibody response. In addition to being surface accessible and conserved across pneumococcal strains the vaccine candidate should also be highly immunogenic. The presence of strong B-cell and T-cell epitopes is important for eliciting a robust adaptive immune response. Linear B-cell epitopes were predicted using the ABCpred and BCpred servers. ABCpred identified 17 epitopes of 16 amino acids in length, with their respective antigenicity scores listed in Table 1 (see PDF). The score reflects an average of multiple parameters, including sequence conservation, similarity to experimentally validated B-cell epitopes, predicted secondary structure and solvent accessibility. Higher scores indicate greater predicted antigenicity. Predictions from BCpred server are presented in Table 2 (see PDF). The BCpred server predicts linear B-cell epitopes based on sequence features such as hydrophilicity, flexibility, surface accessibility, polarity and exposure. A higher prediction score suggests increased likelihood of the epitope being immunogenic. Four of the 5 linear B-cell epitopes predicted by BCpred server were also predicted by ABCpred server with both tools giving high scores. Discontinuous (conformational) B-cell epitopes were predicted using the ElliPro server based on the refined SPD_0792 model and depicted in Figure 2 (see PDF) and listed in Table 3 (see PDF), along with their PI scores. The PI score reflects the relative accessibility of residues. A PI score of 0.9 indicates that the residue lies outside 90% of the ellipsoid volume of the protein, suggesting higher surface exposure. All three tools consistently indicated the presence of multiple strongly antigenic B-cell epitopes, supporting the immunogenic potential of SPD_0792. We predicted the MHC class II-restricted T-cell epitopes present in SPD_0792 using the IEDB analysis resource. Predicted 15-mer epitopes and their percentile ranks are listed in Table 4 (see PDF). Predictions were made for murine MHC-II alleles, including H2-IAb, H2-IAd, H2-IAk, H2-IAq, H2-IAs, H2-IAu, H2-IAb, H2-IEd and H2-IEk. Percentile ranks were calculated by comparing the binding scores to those of 5 million random 15-mer peptides from the SWISSPROT database. Lower percentile ranks correspond to higher binding affinities to MHC class II molecules. Only peptides with a percentile rank of 3.0 or below were included in the table. These data suggest SPD_0792 is highly likely to elicit a very robust adaptive immune response. Murine MHC class-II alleles were chosen for the analysis as the initial experimental validation of the vaccine potential will be done using mouse models of pneumococcal infections.

After having identified a potential vaccine candidate we wanted to express and purify rSPD_0792. The open reading frame encoding SPD_0792 was PCR-amplified from the genomic DNA of *S. pneumoniae* strain D39. The amplicon (928 bp) was cloned into the pET-22b(+) expression vector, and successful cloning was verified by restriction digestion and nucleotide sequencing. rSPD_0792 (~34.4 kDa) was expressed in the soluble fraction with a C-terminal His-tag and purified using Ni-NTA affinity chromatography. The purity of the purified fraction was assessed by SDS-PAGE (Figure 3A - see PDF). The recombinant protein preparation was found to be more than 75% pure. In addition to the rSPD_0792, we observed two minor bands on SDS-PAG. The presence of the His-tag on SPD_0792 was confirmed by immunoblotting with anti-His tag antibodies (Figure 3B - see PDF). We plan to further evaluate this novel candidate for a protein-based pneumococcal vaccine using *in vitro* and animal models of pneumococcal infections, individually or in combination with one or more pneumococcal proteins.

In this study, we used computational tools to predict the subcellular localization and immunogenicity of the pneumococcal protein SPD_0792. Similar in silico methods have been applied to various pathogens. For instance, ROP29 was identified as a potential vaccine target in *T. gondii* through computational analysis [19]. Mir *et al.* predicted B-cell and T-cell epitopes in SARS-CoV-2 using similar approaches [15]. Bioinformatics tools have also supported vaccine design against coxsackievirus [18], *A. baumannii* [16] and *M. tuberculosis* [17].

## Conclusion:

The presence of a lipobox motif indicates that SPD_0792 is a surface-exposed lipoprotein. Further, SPD_0792 contains highly immunogenic B-cell and helper T-cell epitopes capable of inducing a robust adaptive immune response. Moreover, rSPD_0792 was successfully expressed in the soluble fraction. Additional studies using appropriate animal models are required to experimentally validate its potential as a vaccine candidate.
